# Protein-Bound Uremic Toxins Induce Reactive Oxygen Species-Dependent and Inflammasome-Mediated IL-1β Production in Kidney Proximal Tubule Cells

**DOI:** 10.3390/biomedicines9101326

**Published:** 2021-09-26

**Authors:** Milos Mihajlovic, Merle M. Krebber, Yi Yang, Sabbir Ahmed, Valeria Lozovanu, Daria Andreeva, Marianne C. Verhaar, Rosalinde Masereeuw

**Affiliations:** 1 Division of Pharmacology, Utrecht Institute for Pharmaceutical Sciences, Utrecht University, 3584 CG Utrecht, The Netherlands; m.mihajlovic@uu.nl (M.M.); y.yang@uu.nl (Y.Y.); s.ahmed@uu.nl (S.A.); v.lozovanu@erasmusmc.nl (V.L.); daria.andreeva@kcl.ac.uk (D.A.); 2 Department Nephrology and Hypertension, University Medical Center Utrecht, 3508 GA Utrecht, The Netherlands; m.m.krebber-2@umcutrecht.nl (M.M.K.); m.c.verhaar@umcutrecht.nl (M.C.V.)

**Keywords:** chronic kidney disease, protein-bound uremic toxins, indoxyl sulfate, NLRP3 inflammasome, IL-1β, kidney inflammation, oxidative stress, NF-κB, subtotal nephrectomy, conditionally immortalized proximal tubule cells

## Abstract

Protein bound-uremic toxins (PBUTs) are not efficiently removed by hemodialysis in chronic kidney disease (CKD) patients and their accumulation leads to various co-morbidities via cellular dysfunction, inflammation and oxidative stress. Moreover, it has been shown that increased intrarenal expression of the NLRP3 receptor and IL-1β are associated with reduced kidney function, suggesting a critical role for the NLRP3 inflammasome in CKD progression. Here, we evaluated the effect of PBUTs on inflammasome-mediated IL-1β production in vitro and in vivo. Exposure of human conditionally immortalized proximal tubule epithelial cells to indoxyl sulfate (IS) and a mixture of anionic PBUTs (UT mix) increased expression levels of NLRP3, caspase-1 and IL-1β, accompanied by a significant increase in IL-1β secretion and caspase-1 activity. Furthermore, IS and UT mix induced the production of intracellular reactive oxygen species, and caspase-1 activity and IL-1β secretion were reduced in the presence of antioxidant N-acetylcysteine. IS and UT mix also induced NF-κB activation as evidenced by p65 nuclear translocation and IL-1β production, which was counteracted by an IKK inhibitor. In vivo, using subtotal nephrectomy CKD rats, a significant increase in total plasma levels of IS and the PBUTs, kynurenic acid and hippuric acid, was found, as well as enhanced urinary malondialdehyde levels. CKD kidney tissue showed an increasing trend in expression of NLRP3 inflammasome components, and a decreasing trend in superoxide dismutase-1 levels. In conclusion, we showed that PBUTs induce inflammasome-mediated IL-1β production in proximal tubule cells via oxidative stress and NF-κB signaling, suggesting their involvement in disease-associated inflammatory processes.

## 1. Introduction

Chronic kidney disease (CKD) is a pathological condition characterized by a progressive loss of kidney function, leading to end stage kidney disease (ESKD) and the need for kidney replacement therapies. It has a global estimated prevalence of 11–15%, which further confirms its huge impact on both socio-economic aspects and the health system [1,2].

It has been recognized that CKD is accompanied by persistent local and systemic inflammation, characterized by increased production of multiple inflammatory mediators, oxidative stress, acidosis and intestinal dysbiosis [3,4]. The inflammatory processes present in CKD have been shown to contribute to many complications, especially cardiovascular events such as coronary artery calcification, atherosclerosis and heart failure, but also to insulin resistance, endothelial dysfunction, bone disorders, cognitive dysfunction, susceptibility to infections and overall increased CKD mortality [5,6,7,8,9,10,11,12].

Among the main players involved in the development and progression of CKD-associated inflammation are uremic retention solutes [13]. These toxins can be classified into three groups based on their molecular weight and protein binding capacity: (1) small water-soluble molecules, (2) middle molecules and (3) protein-bound molecules, frequently denominated protein-bound uremic toxins (PBUTs) [14,15]. The latter category is particularly troublesome for ESKD patients as these toxins are not easily removed by dialysis treatment due to their high binding capacity to albumin [16]. Many PBUTs, including indoxyl sulfate (IS) and *p*-cresyl sulfate, find their origin in the gut as products of microbial metabolism and have been shown to be involved in the development of CKD-related co-morbidities, mostly by mediating pro-inflammatory and oxidative processes [17,18].

The inflammasome is one of the inflammation-related mechanisms that recently has gained a lot of attention for its pathogenic role in kidney diseases [19]. These large multiprotein intracellular complexes are in most cases comprised of a sensor protein, typically a member of the nucleotide-binding oligomerization domain (NOD)-like receptor (NLRs), the absent in melanoma 2 (AIM2)-like receptor (ALRs) or the retinoic acid-inducible gene-I (RIG-I)-like receptor (RLRs) families, the adaptor protein apoptosis-associated speck-like protein containing a caspase recruitment domain (ASC) and caspase-1. These are assembled and activated in the presence of danger and stress-associated stimuli leading to caspase-1 activation and subsequent pro-inflammatory interleukin-1β (IL-1β) and interleukin-18 (IL-18) release and pyroptosis [20].

One of the most studied inflammasomes is NLRP3 that usually requires two signals for its activation: the first one to induce transcription of pro-IL-1β and responsiveness of NLRP3 to the second signal that triggers its activation [21]. These signals include various pathogen-associated molecular patterns (PAMPs) and damage-associated molecular patterns (DAMPs), such as ATP, uric acid and calcium pyrophosphate dehydrate (CPPD) crystals and amyloid-β fibrils [22,23,24,25,26,27]. Although not fully elucidated as of yet, the main proposed mechanisms of NLRP3 inflammasome activation include the ion channel model, the lysosome rupture model, and the reactive species (ROS) model. The latter one proposes that ROS cause dissociation of thioredoxin/thioredoxin-interacting protein (TXNIP) complex, and TXNIP subsequently binding to and activation of NLRP3 [28,29].

Based on the findings that the inflammasome plays a role in CKD pathogenesis and progression and the fact that PBUTs have a prominent pro-inflammatory capacity, we hypothesized that this group of uremic toxins are drivers of inflammasome-related inflammation within the kidney proximal tubule. This is an important segment of the nephron because of its role in PBUTs uptake and active tubular secretion and its susceptibility to PBUTs’ toxic effects [30,31]. The present work was designed to study the effects of IS, a representative PBUT, and a mixture of several anionic PBUTs [32] (UT mix; Table 1) on NLRP3 inflammasome activation. Given that the organic anion transporter 1 (OAT1) is an important transporter responsible for the uptake of anionic uremic toxins into proximal tubule cells thus contributing to their removal, previously established and characterized human conditionally immortalized proximal tubule epithelial cells overexpressing OAT1 (ciPTEC-OAT1) were employed for in vitro studies [32,33,34,35].

In addition, the link between PBUTs and kidney tissue-specific expression of inflammasome-related components in a rat subtotal nephrectomy (SNX) model was evaluated in vivo. We examined the effect of IS and UT mix in vitro on the expression of all inflammasome components (NLRP3, ASC, caspase-1 and IL-1β) in ciPTEC-OAT1, on caspase-1 activity and IL-1β secretion, as well as their ability to induce oxidative stress. In addition, we evaluated the role of NF-κB signaling pathway as the priming signal in PBUTs-mediated inflammasome activation. Finally, the expression of NLRP3, caspase-1 and IL-1β in kidney tissue, plasma levels of various PBUTs and oxidative status in the CKD animal model were assessed. Determining the role of PBUTs in inflammasome activation in the proximal tubule and unravelling the mechanisms by which this occurs could lead to new intervention modalities aimed at reducing inflammation-mediated kidney injury.

## 2. Materials and Methods

### 2.1. Reagents

All reagents were purchased from Sigma-Aldrich (Zwijndrecht, The Netherlands) unless stated otherwise. In the present study, lipopolysaccharide (LPS) from *Escherichia coli* 0127:B8 was used. The toxins, *p*-cresyl glucuronide (pCG) and *p*-cresyl sulfate (pCS), were synthesized by the Institute for Molecules and Materials, Radboud University, Nijmegen, The Netherlands, as previously described [36]. IκB kinase (IKK) inhibitor BMS-345541 was obtained from Axon Medchem (Groningen, The Netherlands). Water (LC-MS grade), acetonitrile (ACN; HPLC-S grade) and methanol (HPLC grade) were obtained from Biosolve (Valkenswaard, The Netherlands). Formic acid (analytical grade) was purchased at Merck (Darmstadt, Germany). Ultrapure water was produced by a Milli-Q^®^ Advantage A10 Water Purification Systems (Merck, Schiphol-Rijk, The Netherlands). Cell culture plates were obtained from Greiner Bio-One (Monroe, NC, USA).

### 2.2. CiPTEC-OAT1 Cell Culture

The ciPTEC-OAT1 cell line was cultured in Dulbecco’s Modified Eagle Medium/Nutrient Mixture F-12 (1:1 DMEM/F-12) (Gibco, Life Technologies, Paisley, UK) supplemented with 10% fetal calf serum (FCS) (Greiner Bio-One, Alphen aan den Rijn, The Netherlands), 5 μg/mL insulin, 5 μg/mL transferrin, 5 μg/mL selenium, 35 ng/mL hydrocortisone, 10 ng/mL epidermal growth factor and 40 pg/mL tri-iodothyronine, without addition of antibiotics and up to a maximum of 60 passages, as reported previously [35]. Cells were kept in culture at 33 °C and 5% (*v/v*) CO_2_. Prior to the experiments, cells were seeded at a density of 55,000 cell/cm^2^, grown for one day at 33 °C, 5% (*v/v*) CO_2_, and cultured for 7 days at 37 °C, 5% (*v/v*) CO_2_ to allow maturation, refreshing the medium every other day.

### 2.3. CiPTEC-OAT1 Exposure to PBUTs

IS concentrations used for treatment of ciPTEC-OAT1 were: 200 μM, 1 mM and 2 mM. PBUTs mixture (UT mix) was prepared as described previously [32] with slightly adjusted concentrations (Table 1) to replicate PBUTs plasma levels observed in CKD patients. The mixture was prepared as a 100× solution and subsequently diluted to 1× or 2.5× for treatment of ciPTEC-OAT1.

### 2.4. Caspase-1 Activity

To measure caspase-1 activity, ciPTEC-OAT1 cells were exposed to desired treatment conditions for 2 h (in case of LPS/ATP co-treatment, ATP was added during the final 30 min of exposure) and incubated at 37 °C, 5% (*v/v*) CO_2_, in the absence or presence of specific inhibitors ac-YVAD-cmk or N-acetyl cysteine (NAC; both added 30 min prior to the start of the treatments). Following incubation, caspase-1 activity was determined using Caspase-Glo^®^ 1 inflammasome assay kit (Promega, Leiden, The Netherlands) according to manufacturer’s instructions. Luminescence was measured using the Fluoroskan Ascent FL microplate reader (Thermo Fisher Scientific, Vantaa, Finland). Measured luminescence values were corrected for background and used to calculate relative caspase-1 activity, using untreated cells as the reference.

### 2.5. Enzyme-Linked Immunosorbent Assay (ELISA)

The production of IL-1β was measured using an Enzyme-Linked Immunosorbent Assay (ELISA). Cell culture supernatants were collected after 24 h of the treatments, in the absence or presence of specific inhibitors. Afterwards, cell culture supernatants were centrifuged for 10 min, 240× *g*, 4 °C and stored at −20 °C until analyzed. DuoSet^®^ ELISA Development Systems kit (Human IL-1 beta/IL-1F2 #DY201; R&D Systems, Abingdon, UK) was used to quantify IL-1β levels in cell culture supernatants following manufacturer’s instructions. The optical density was determined using the iMark Microplate Absorbance Reader (Bio-Rad Laboratories, Hercules, CA, USA) set to 450 nm. Each sample was measured in duplicates and quantification was done using Microplate Manager software (version 6.0, Bio-Rad Laboratories, Hercules, CA, USA), generating a four-parameter logistic (4-PL) curve-fit.

### 2.6. RNA Extraction, cDNA Synthesis and Real-Time PCR

Total RNA from ciPTEC-OAT1 exposed to uremic toxins or LPS/ATP co-treatment, in the absence or presence of BMS-345541 (added 1 h prior to the treatments), for either 4 h or 24 h, was isolated using the RNeasy Mini kit (Qiagen, Venlo, The Netherlands) following the manufacturer’s instructions and quantified using the NanoDrop^®^ ND-1000 spectrophotometer (Thermo Fisher Scientific, Wilmington, DE, USA). The synthesis of cDNA was performed using 800 ng of total mRNA per sample and by means of the iScriptTM Reverse Transcription Supermix (Bio-Rad Laboratories, Hercules, CA, USA) and T100™ Thermal Cycler (Bio-Rad Laboratories, Hercules, CA, USA). Subsequently, Real-Time PCR was performed using the iQ SYBR^®^ Green Supermix (Bio-Rad Laboratories, Hercules, CA, USA) as indicated in manufacturer’s protocol and by means of CFX96TM Real-Time PCR Detection System (Bio-Rad Laboratories, Hercules, CA, USA). The data were analyzed using Bio-Rad CFX ManagerTM Software version 3.1 (Bio-Rad Laboratories, Hercules, CA, USA) and expressed as relative gene expression, using untreated cells as the reference sample. *HPRT1* and *RPS13* were used as housekeeping genes for normalization. Specific sense and anti-sense primers for *HPRT1* (forward: ACATCTGGAGTCCTATTGACATCG; reverse: CCGCCCAAAGGGAACTGATAG), *RPS13* (forward: GCTCTCCTTTCGTTGCCTGA; reverse: ACTTCAACCAAGTGGGGACG), *NLRP3* (forward: GATCTTCGCTGCGATCAACA; reverse: GGGATTCGAAACACGTGCATTA), *PYCARD* (ASC; forward: CAGCAACACTCCGGTCAG; reverse: AGCTGGCTTTTCGTATATTGTG), *CASP1* (caspase-1; forward: GCCTGTTCCTGTGATGTGGAG; reverse: TGCCCACAGACATTCATACAGTTTC), *IL1B* (IL-1β; forward: TCGCCAGTGAAATGATGGCT; reverse: TGGAAGGAGCACTTCATCTGTT), *HMOX1* (heme oxygenase 1; forward: GACCCATGACACCAAGGACC; reverse: TCCACGGGGGCAGAATCTTG), *NFKBIA* (NFκB inhibitor alpha; forward: AATGCTCAGGAGCCCTGTAA; reverse: CTGTTGACATCAGCCCCACA) and *TNF* (TNF-α; forward: TGTTGTAGCAAACCCTCAAGC; reverse: TATCTCTCAGCTCCACGCCA) were synthesized by Biolegio (Nijmegen, The Netherlands).

### 2.7. Intracellular Reactive Oxygen Species (ROS) Detection

Intracellular ROS generation was measured using chloromethyl-2′,7′-dichlorofluorescein diacetate (CM-H_2_DCFDA; #C6827; Invitrogen, Carlsbad, CA, USA) and following the manufacturer’s instructions. Briefly, cells were washed once with Hank’s Balanced Salt Solution (HBSS; Gibco, Life Technologies, Paisley, UK), immediately loaded with CM-H_2_DCFDA (10 μM in HBSS) and incubated at 37 °C, 5% (*v/v*) CO_2_, in the dark for 25 min. Afterwards, cells were washed with a complete culture medium and left to recover for 15 min. Next, cells were exposed to various concentrations of uremic toxins, in the absence or presence of antioxidant agents, NAC (1.8 mM) or 6-hydroxy-2,5,7,8-tetramethylchroman-2-carboxylic acid (Trolox; 100 μM), for 2 h at 37 °C, 5% (*v/v*) CO_2_, in the dark. Hydrogen peroxide (H_2_O_2_; 200 µM) was used as a positive control. Following the incubation, fluorescence was measured at an excitation wavelength of 492 nm and emission wavelength of 518 nm, using a fluorescent microplate reader (Fluoroskan Ascent FL, Thermo Fisher Scientific, Vantaa, Finland). Fluorescence intensities measured were corrected for the blank sample (non-stained cells) and used to calculate relative intracellular ROS production, using untreated cells as the reference.

### 2.8. Protein Extraction and Quantification

After exposing cells for 24 h to different concentrations of uremic toxins or LPS/ATP combinatory treatment, cell lysates were obtained by washing the cells twice with cold HBSS and incubating them on ice for 5 min with 350 µL cold RIPA lysis buffer (Thermo Scientific, Rockford, IL, USA) containing 1% (*v/v*) protease inhibitor (Halt Protease Inhibitor Cocktail; Thermo Scientific, Rockford, IL, USA). The lysates were then gathered using a cell scraper and centrifuged at 14,000× *g*, at 4 °C for 15 min. The supernatants were collected for further analyses.

Snap-frozen kidney tissues were dissected on ice into small pieces weighing between 10 and 20 mg, washed once in ice-cold PBS, incubated in ice-cold RIPA buffer (approx. 500 μL per 10 mg of tissue) for 20 min and beads-homogenized thoroughly. The lysed homogenates were then centrifuged at 14,000× *g*, at 4 °C for 15 min and the supernatants were collected for further analyses. The total protein content was determined by using Pierce™ BCA Protein Assay Kit (Thermo Scientific, Waltham, MA, USA) as indicated in manufacturer’s protocol and the samples were stored at −80 °C until further usage.

### 2.9. SDS-PAGE and Western Blot Analysis

The protein samples were denatured and reduced in Laemmli Sample Buffer 4× (Bio-Rad Laboratories, Hercules, CA, USA) containing 2% β-mercaptoethanol for 5 min at 95 °C using the Eppendorf ThermoMixer^®^ C instrument (Eppendorf, Hamburg, Germany). A total 15 µg of proteins per sample were separated using the Mini-PROTEAN^®^ TGX™ 4–20% precast gels by the means of sodium dodecyl sulfate polyacrylamide gel electrophoresis (SDS-PAGE; Bio-Rad Laboratories, Hercules, CA, USA) for 90 min at 100 V and subsequently transferred to a 0.2 µm nitrocellulose membrane (Bio-Rad Laboratories, Hercules, CA, USA) using Trans-Blot^®^ Turbo™ Transfer Pack (Bio-Rad Laboratories, Hercules, CA, USA) and Trans-Blot^®^ Turbo™ Transfer System (Bio-Rad Laboratories, Hercules, CA, USA) for 30 min at 25 V.

Afterwards, the membranes were blocked for 20 min with 5% fat-free dry milk (Nutricia Protifar, Fulda, Germany) in PBS/0.1% Tween-20 (PBS-T) and probed overnight at 4 °C with following primary antibodies, depending on the protein being detected: rabbit anti-NLRP3 (1:250; D4D8T, Cell Signaling Technology, Leiden, The Netherlands), rabbit anti-NLRP3 (1:500; EPR23094-1, Abcam, Amsterdam, The Netherlands), rabbit anti-caspase-1 (1:250; Enzo Life Sciences, Raamsdonksveer, The Netherlands), rabbit anti-IL-1β (1:250; Abcam, Amsterdam, The Netherlands), rabbit anti-SOD-1 (1:50,000; EP1727Y, Abcam, Amsterdam, The Netherlands), mouse anti-ASC (1:500; Santa Cruz Biotechnology, Dallas, TX, USA), mouse anti-β-actin (1:5000; Novus Biologicals, Abingdon, UK), mouse anti-β-actin (1:1000; mAbcam 8226, Abcam, Amsterdam, The Netherlands) and mouse anti-α-tubulin (1:1000; Abcam, Amsterdam, The Netherlands). The membranes were then probed with one of the following HRP-conjugated secondary antibodies: goat anti-rabbit (1:5000; Dako, Carpinteria, CA, USA), or rabbit anti-mouse (1:5000; Dako, Carpinteria, CA, USA). Finally, chemiluminescence was developed with Clarity™ Western ECL Substrate (Bio-Rad Laboratories, Hercules, CA, USA) and the images acquired using the ChemiDoc™ MP Imaging System (Bio-Rad Laboratories, Hercules, CA, USA). The data were analyzed using the Image Lab software (version 5.2, Bio-Rad Laboratories, Hercules, CA, USA), and the imaged bands were quantified by densitometric analysis using ImageJ software version 1.49 (National Institutes of Health, Bethesda, MD, USA). The results are presented as relative protein expression normalized against β-actin or α-tubulin.

### 2.10. Immunocytochemistry

To assess the nuclear expression levels of p65, ciPTEC-OAT1 monolayers pre-incubated with BMS-345541 for 1 h were treated with uremic toxins, LPS or TNF-α for 1 h, then washed with warm HBSS twice and fixated with 4% (*v/v*) paraformaldehyde (PFA) in PBS for 10 min. After additional three washing steps with PBS-T, cells were permeabilized with 0.3% (*v/v*) Triton-X for 10 min. Following three more washing steps in PBS-T, cells were incubated for 30 min in a blocking solution (2% (*v/v*) FCS, 2% (*w/v*) bovine serum albumin (BSA), 0.1% (*v/v*) Tween-20 in HBSS) and exposed to primary antibody rabbit anti-NF-κB p65 (Invitrogen, Carlsbad, CA, USA), diluted in blocking buffer (1:100) and incubated for 1 h at room temperature. Following three washing steps with PBS-T, the secondary antibody donkey anti-rabbit IgG AlexaFluor 488 (Life Technologies, Eugene, OR, USA) was added in a concentration of 1:250 and incubated for 1 h at room temperature. Finally, cells were washed three more times with PBS-T and imaged using a Cell Voyager 7000 (CV7000) confocal microscope (Yokogawa Electric Corporation, Tokyo, Japan) with 20× magnification. At least 3 fields per condition were analyzed using ImageJ software version 1.49 (National Institutes of Health, Bethesda, MD, USA). In total, at least 300 nuclei per condition were used for quantification and statistical analysis. Data were normalized to untreated control and presented as relative expression.

### 2.11. Experimental Animals

Female outbred Sprague Dawley rats (SD, Envigo, Horst, The Netherlands), aged 8 weeks and weighing approximately 200 g were used and housed socially (up to *n* = 4 per cage) under standard climate-controlled conditions with *ad libitum* access to water and food. Animals were acclimated for 7 days prior to start of the study. The study protocol was approved by the Animal Ethics Committee of the University of Utrecht (CCD; AVD115002015310) and in agreement with the current Dutch law on animal experiments. Animals were matched between healthy Sham and CKD groups based on baseline measurements of proteinuria, creatinine, urea and systolic blood pressure (SBP).

### 2.12. CKD Model in Rats

Animals were used as part of a pilot to monitor progression to CKD, using an altered protocol of our previously published surgical 5/6th nephrectomy (SNX) [37]. A week before the SNX, baseline measurements to determine systolic blood pressure (SBP), proteinuria, plasma urea, urine and plasma creatinine were taken. Animals were stratified and randomly assigned to either Sham or CKD group. 

In short, CKD was induced by removing the whole left kidney and polectomy (2/3rd) of the right kidney using retroperitoneal incisions in animal weighing over 200 g in a single procedure. In Sham animals, both kidneys were externalized, the renal capsule was removed and the intact kidneys were placed back. Surgery was performed under isoflurane gas anesthesia (4% induction, 2.5% maintenance and O_2_). The incisions were closed by suturing the muscle layers with continuous stitches (5.0 Vicryl—Ethicon V303H, Lidingö, Sweden) and the skin with continuous intracutaneous stitches (4.0 Vicryl—Ethicon V392H, Lidingö, Sweden). Postoperative buprenorphine (Temgesic, 0.03 mg/kg) was given subcutaneously for a period of 48 h and rats were kept individually overnight on heat pads to recover. 

Rats were subsequently weighed each week and longitudinal measurements were taken every other week to follow CKD progression. SBP was measured by tail cuff sphygmomanometer (LE 5002 LETICA^®^, Panlab, Barcelona, Spain). To determine proteinuria, 24 h urine samples were collected during which rats were placed in metabolic cages without food but with free access to water with 2% glucose. Urine was collected in antibiotic/antimycotic solution (A5955; Sigma, St Louis, MO, USA) and stored at −80 °C. Blood samples were collected from the tail vein into EDTA Microtainers (#365974; BD, Vianen, The Netherlands). Urinary protein levels were measured by Bradford Assay (Bio-Rad Laboratories, Hercules, CA, USA). Sodium and potassium levels were determined by flame photometry. Plasma and urinary urea were determined by DiaSys Urea CT FS (DiaSys Diagnostic Systems, Holzheim, Germany). Plasma and urinary creatinine levels were determined by DiaSys Creatinine PAP FS kit (DiaSys Diagnostic Systems, Holzheim, Germany). 

Animals were sacrificed when humane endpoints (HEPs), as determined beforehand, were reached. HEPs included progressive proteinuria (>400 mg/24 h for any given measurement), progressive uremia (>20 mmol/L for any given measurement), progressive weight loss (>15% within 7 days) or a cumulative decreased welfare score based on the rat grimace scale (Intensity >4 as visually determined by two independent observers) [38]. CKD and age-matched Sham animals were sacrificed by exsanguination through cardiac punction under isoflurane anesthesia. Terminal blood was collected, and kidneys were dissected and subsequently snap-frozen (−80 °C) for later analysis or fixed in 4% PFA and embedded in paraffin for sectioning.

### 2.13. Immunohistochemistry

Tubulo-interstitial (TI) damage was scored on periodic-acid Schiff (PAS) 3 μm thick stained slides [39]. After incubation with 1% periodic acid, slides were stained in Schiffs reagent in the dark and counterstained with haematoxylin. 

In short, TI was scored on a scale from 0–5 and independently defined as inflammatory cell infiltrate, tubular atrophy, dilatation or interstitial fibrosis. At least 10 non-overlapping field were analyzed by an experienced researcher blinded to group allocation. 

Pan-macrophages were stained using ED1^+^ (monocyte/macrophage marker; mouse anti-rat CD68, ab31630, 1:250; Abcam, Cambridge, UK). 3 μm deparaffinized sections were first blocked with 5% hydrogen peroxide in PBS and subsequently subjected to indirect heat-induced antigen retrieval (TRIS/EDTA, pH 9.0). After blocking with Superblock^TM^ (Thermo Fischer Scientific, San Jose, CA, USA), the primary antibody was incubated for 1 h at room temperature. Sections were covered with entellan after incubation with secondary horseradish peroxidase (HRP)-conjugated goat-anti-mouse IgG (Brightvision Immunologic, Duiven, The Netherlands) visualization with Nova Red and haematoxylin counterstain. To analyze ED1^+^ cells, least 50 glomeruli and 20 non-overlapping tubular fields were manually counted. Pictures were taken on a BX-51 microscope (Olympus, Leiderdorp, The Netherlands).

### 2.14. Thiobarbituric Acid Reactive Substances (TBARS) Assay for Lipid Peroxidation Detection

Lipid peroxidation end-product, malondialdehyde (MDA), was quantified in urine samples using Lipid peroxidation (MDA) assay kit (Sigma-Aldrich, Zwijndrecht, The Netherlands) and following instructions as described in manufacturer’s protocol. Values measured were corrected for background and used to calculate MDA levels, expressed as nmol/mL. 

### 2.15. PBUTs Plasma Levels Quantification by LC-MS

An Accela LC system (quaternary pump and autosampler) coupled to a TSQ Quantum Ultra triple quadrupole mass spectrometer with heated electrospray ionization (ESI) was used for this study. Equipment and software for controlling, data recording and processing (Xcalibur version 2.07) were supplied by Thermo Fischer Scientific (San Jose, CA, USA). A Waters ACQUITY UPLC HSS T3 column (100 mm × 2.1 mm, 1.8 µm particles) combined with an ACQUITY UPLC HSS T3 VanGuard pre-column (5 mm × 2.1 mm, 1.8 µm particles) was used and kept at a temperature of 40 °C. Mixed isocratic and gradient elution at a flow rate of 0.45 mL/min was applied for analyte separation. In the mobile phase, solvent A consisted of 0.2% (*v*/*v*) acetic acid, and solvent B was ACN. The final method was an initial isocratic composition of 5% B held for 1 min, followed by a linear increase to 15% B for the next 1 min, and to 20% B in another 1 min. Then, B was increased linearly to 80% over the next min to flush the column. To re-equilibrate, B was reduced back to the initial isocratic composition of 5% and was held for 2 min.

Plasma samples were processed prior to LC-MS analysis. A volume of 20 µL plasma or surrogate matrix was pipetted into a 1.5 mL Eppendorf tube (Eppendorf, Hamburg, Germany). Ultra-pure water was used as a surrogate matrix for calibration curve and quality control samples. For the calibration curve and quality control samples, the 20 µL water sample contained the standards. Next, 30 µL of cold (4 °C) ACN with internal standards d4-kynurenine, d5-kynurenic acid, 13C6-indoxyl sulfate, d5- indole-3-acetic acid, d7-*p*-cresyl sulfate, d7-*p*-cresyl glucuronide and d5-hippuric acid were added. Samples were vortexed for 5 min on a plate shaker and centrifuged for 5 min at 4000 rpm. A 35 µL sample of the supernatant was then collected in 1 mL round-bottom well of a polypropylene 96-deep well plate and diluted with 200 µL of ultra-pure water. Finally, the plate was gently shaken before placing in the autosampler for analysis. Analyzed data are expressed as ng/mL or µg/mL. 

### 2.16. Data Analysis

All data are presented as mean ± standard error of the mean (SEM). Statistical analysis was performed using one-way or two-way ANOVA followed by Dunnett’s and Sidak’s multiple comparison tests respectively, or where appropriate, an unpaired *t*-test. A *p*-value < 0.05 was considered significant and indicated using one asterisk. Software used for statistical analysis was GraphPad Prism (version 8.43; GraphPad software, La Jolla, CA, USA). Most experiments were repeated independently at least three times and in duplicates, unless otherwise stated. The exact sample size for each experiment is indicated in the corresponding figure legend.

## 3. Results

### 3.1. CiPTEC-OAT1 Respond to NLRP3 Inflammasome-Activating Stimuli

**Figure 1:**
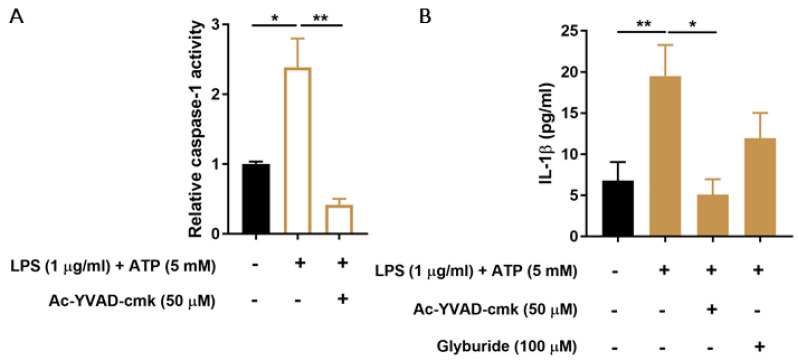
Effect of LPS and ATP on caspase-1 activity and IL-1β production in ciPEC-OAT1. (**A**) Relative caspase-1 activity in ciPTEC-OAT1 upon exposure to LPS (1 μg/mL; 2 h) and ATP (5 mM; 30 min) in the absence or presence of specific caspase-1 inhibitor, ac-YVAD-cmk (50 μM). (**B**) IL-1β secreted levels (pg/mL) by ciPEC-OAT1 following 24 h treatment with LPS (1 μg/mL; 24 h) and ATP (5 mM; 30 min), in the absence or presence of inhibitors ac-YVAD-cmk (50 μM) or glyburide (100 μM). Data are derived from three independent experiments performed in duplicate and expressed as mean ± SEM. * *p* < 0.05, ** *p* < 0.01 (One-way ANOVA followed by Dunnett’s multiple comparison test, using as LPS/ATP treated cells as a control).

Known activating stimuli of the NLRP3 inflammasome, LPS and ATP, were used to determine whether ciPTEC-OAT1 cells respond with inflammasome activation, as evaluated by caspase-1 activity and IL-1β secretion. As shown in Figure 1A, 24 h exposure to LPS and exposure to ATP in the last 30 min of stimulation significantly increased caspase-1 activity, which was drastically reduced in the presence of a known inhibitor of caspase-1 function, ac-YVAD-cmk. Similarly, IL-1β secretion was increased upon LPS and ATP stimulation (Figure 1B). Specific inhibitors of caspase-1 activity (ac-YVAD-cmk) and of an ATP-sensitive potassium channel and NLRP3 (glyburide), reduced IL-1β secreted levels by approximately 75% and 39%, respectively.

### 3.2. PBUTs Induce Expression of NLRP3 Inflammasome-Related Genes in Proximal Tubule Cells 

**Figure 2:**
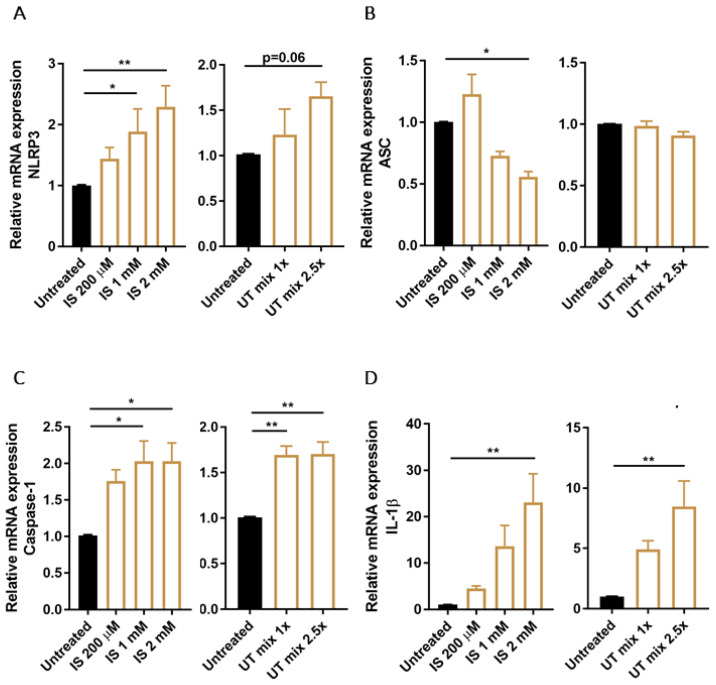
Expression of NLRP3 inflammasome related genes in ciPTEC-OAT1 after exposure to PBUTs. Relative mRNA expression of (**A**) NLRP3, (**B**) ASC, (**C**) caspase-1 and (**D**) IL-1β upon exposure to either IS (200 μM, 1 mM, 2 mM) or UT mix (1×, 2.5×), compared to control (untreated ciPTEC-OAT1). Data are derived from three independent experiments performed in duplicate and expressed as mean ± SEM. * *p* < 0.05, ** *p* < 0.01 (One-way ANOVA followed by Dunnett’s multiple comparison test, using untreated cells as a control).

Next, we determined the expression levels of the NLRP3 inflammasome and its related components, ASC, caspase-1 and IL-1β, in ciPTEC-OAT1 upon exposure to PBUTs. The data show that IS was responsible for a dose-dependent increase of NLRP3 expression, with more than 2-fold increase at the highest concentration tested (Figure 2A). ASC expression levels, on the other hand, were reduced by IS while UT mix did not affect ASC mRNA levels (Figure 2B). Further, caspase-1 was upregulated after IS and UT mix stimulation (Figure 2C) as well as IL-1β expression levels in a dose-dependent manner (Figure 2D). Control treatment with LPS and ATP showed similar profiles of ASC, caspase-1 and IL-1β expression levels, while NLRP3 expression was reduced by more than 50% (Figure S1A–D).

### 3.3. PBUTs Induce Expression of NLRP3 Inflammasome-Related Proteins

**Figure 3:**
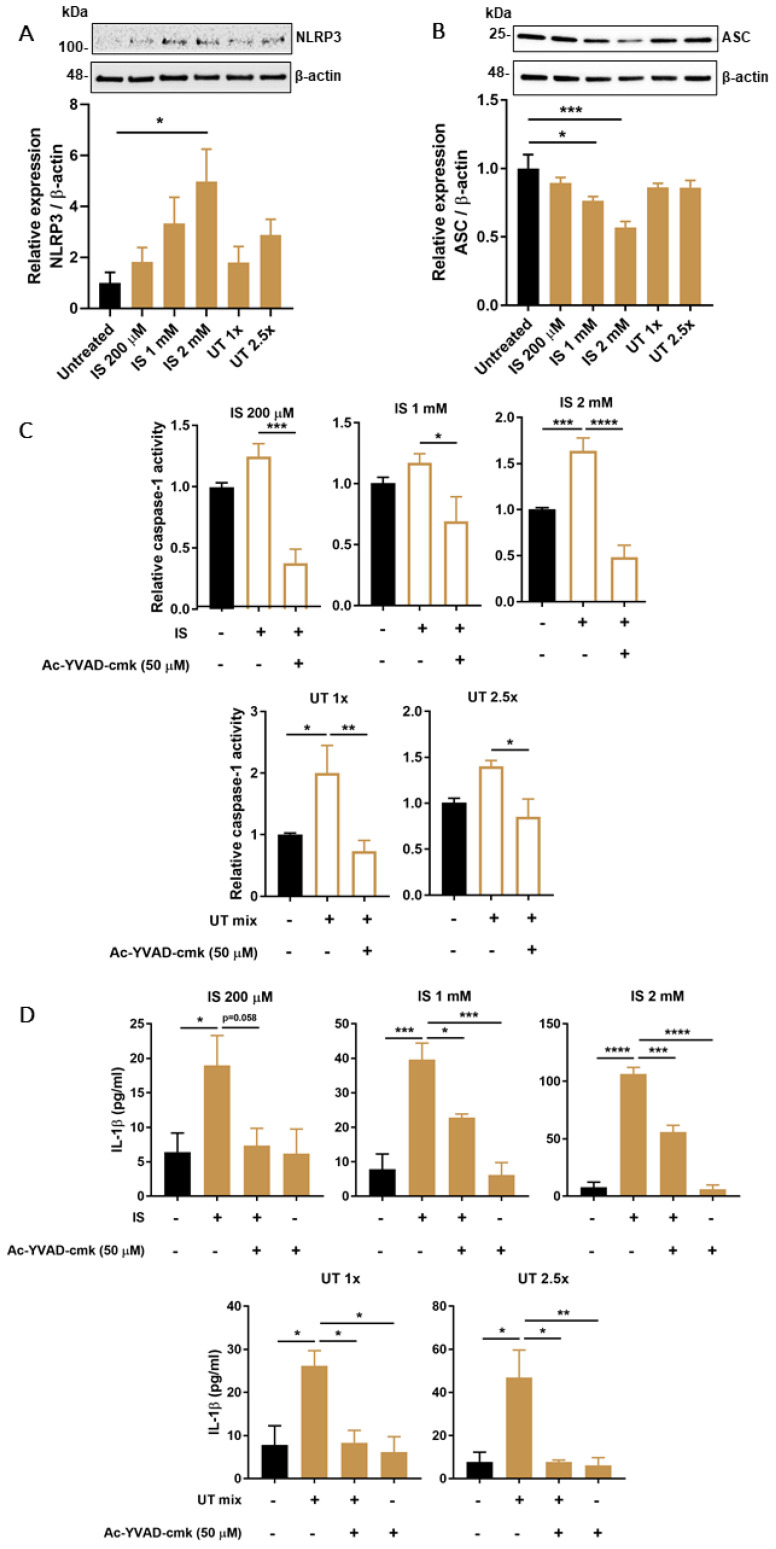
Effect of PBUTs on protein levels or functional activity of NLRP3 inflammasome-related components in ciPTEC-OAT1. Representative Western blot and quantification of relative expression of (**A**) NLRP3 and (**B**) ASC in ciPTEC-OAT1 following 24 h exposure to IS (200 μM, 1 mM, 2 mM) and UT mix (1×, 2.5×); normalized to β-actin. (**C**) Relative caspase-1 activity in ciPTEC-OAT1 upon 2 h exposure to IS (200 μM, 1 mM, 2 mM) and UT mix (1×, 2.5×), in the absence or presence of specific caspase-1 inhibitor, ac-YVAD-cmk (50 μM). (**D**) IL-1β secreted levels (pg/mL) by ciPEC-OAT1 upon 24 h treatment with IS (200 μM, 1 mM, 2 mM) and UT mix (1×, 2.5×), in the absence or presence of ac-YVAD-cmk (50 μM). Data are derived from at least three independent experiments performed in triplicate and expressed as mean ± SEM. * *p* < 0.05, ** *p* < 0.01, *** *p* < 0.001, **** *p* < 0.0001 (One-way ANOVA followed by Dunnett’s multiple comparison test, using either untreated or PBUTs treated cells as a control).

Following 24 h exposure to increasing concentrations of IS or UT mix, NLRP3 levels showed a dose-dependent increase at all tested concentrations (Figure 3A). Similar to what was observed for mRNA levels, ASC protein expression was downregulated in a dose-dependent manner after exposure to IS, while UT mix did not have a significant impact (Figure 3B). Treatment with LPS and ATP also did not significantly affect NLRP3 or ASC levels (Figure S1E,F). Caspase-1 activity was slightly increased after both IS and UT mix treatments at all concentrations evaluated, which was counteracted by ac-YVAD-cmk treatment (Figure 3C). One of the main indicators of inflammasome activation, IL-1β secretion, was also increased at all concentrations of IS and UT mix tested, which was reduced in the presence of ac-YVAD-cmk (Figure 3D).

### 3.4. PBUTs Induce Oxidative Stress and ROS-Dependent Caspase-1 Activity and IL-1β Production

**Figure 4:**
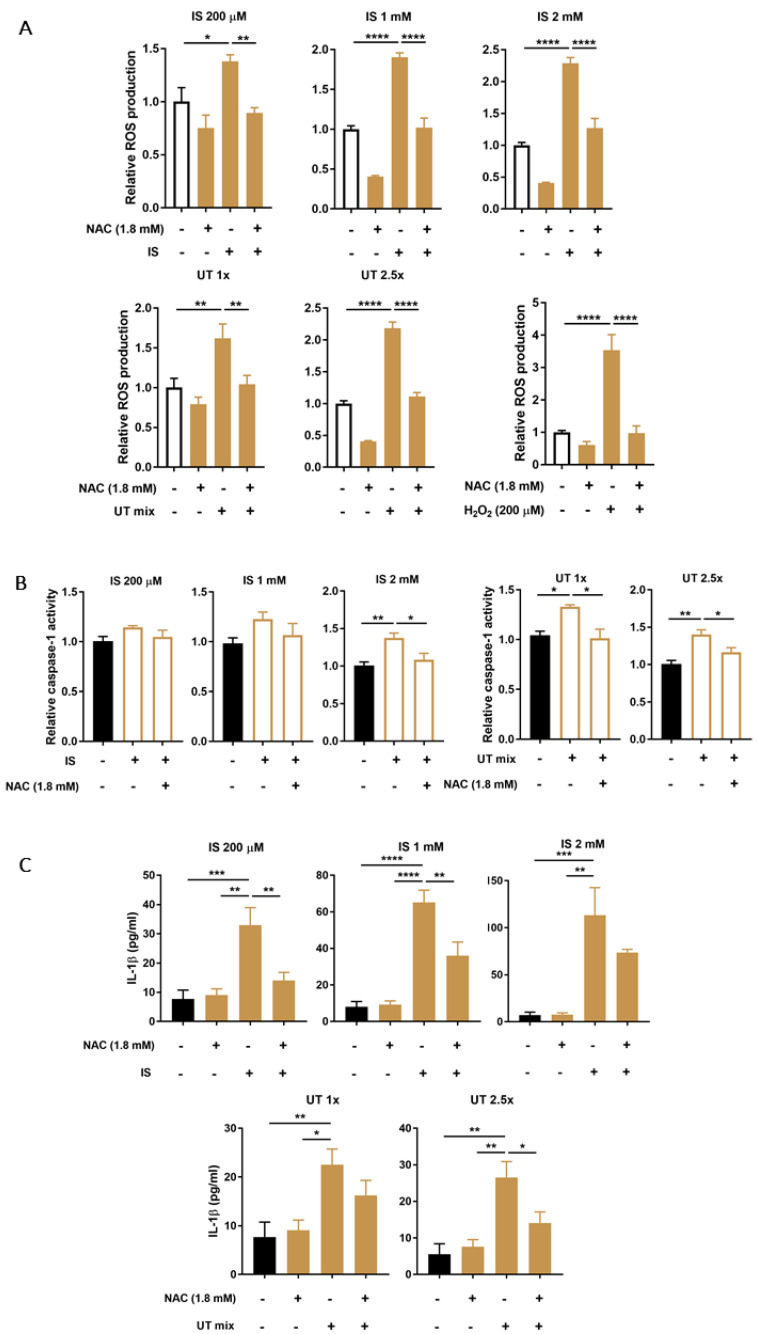
Effect of PBUTs-induced oxidative stress on caspase-1 activity and IL-1β production in ciPTEC-OAT1. (**A**) Relative intracellular ROS production in ciPTEC-OAT1 after 2 h exposure to IS (200 μM, 1 mM, 2 mM), UT mix (1×, 2.5×) and H_2_O_2_ (200 μM, positive control), in the absence or presence of ROS inhibitor NAC (1.8 mM). (**B**) Relative caspase-1 activity in ciPTEC-OAT1 following 2 h exposure to IS (200 μM, 1 mM, 2 mM) and UT mix (1×, 2.5×), in the absence or presence of NAC (1.8 mM). (**C**) IL-1β secreted levels (pg/mL) by ciPEC-OAT1 upon 24 h treatment with IS (200 μM, 1 mM, 2 mM) and UT mix (1×, 2.5×), in the absence or presence of NAC (1.8 mM). Data are derived from at least three independent experiments performed in triplicate and expressed as mean ± SEM. * *p* < 0.05, ** *p* < 0.01, *** *p* < 0.001, **** *p* < 0.0001 (One-way ANOVA followed by Dunnett’s multiple comparison test, using PBUTs treated cells as a control).

To evaluate the effects of PBUTs on oxidative stress, which is a known trigger of NLRP3 inflammasome activation, the intracellular production of ROS was measured following 2 h exposure to either increasing concentrations of IS or UT mix. Results indicate a clear increase of ROS production in ciPTEC-OAT1 after exposures, with H_2_O_2_ serving as positive control (Figure 4A). In all conditions tested, ROS were reduced to almost baseline levels in the presence of NAC (Figure 4A) or Trolox (Figure S2A) as antioxidant agents. 

To determine the role of oxidative stress on the inflammasome activation, caspase-1 activity and IL-1β secretion were evaluated after treatment with PBUTs in the absence or presence of antioxidant NAC. As shown in Figure 4B, lower concentrations of IS caused an increasing trend in caspase-1 activity, while high IS concentrations induced a significant increase in caspase-1 activity that was abolished to baseline levels in the presence of NAC. UT mix significantly increased caspase-1 activity that was counteracted by NAC. The secreted levels of IL-1β were also affected by PBUTs-mediated oxidative stress (Figure 4C). IS treatments increased IL-1β levels, which were significantly reduced when NAC was present as a co-treatment. NAC was also able to counteract the UT mix-mediated IL-1β increased levels. A similar, although less pronounced, pattern of IL-1β secretion was observed when Trolox was used as antioxidant for the co-treatment with PBUTs (Figure S2B).

### 3.5. PBUTs Activate NF-κB Leading to IL-1β Production

**Figure 5:**
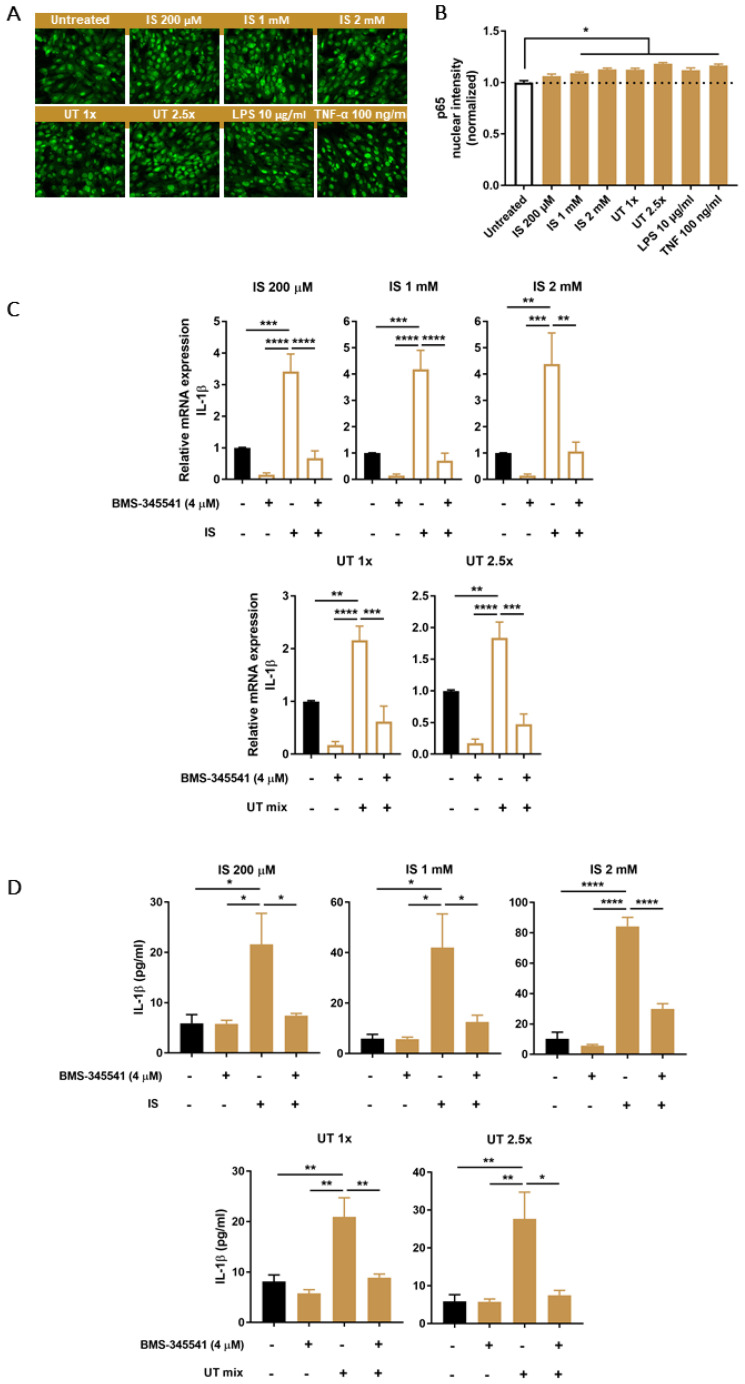
Effect of PBUTs on NF-κB signaling and the role of NF-κB on PBUTs-mediated caspase-1 activity and IL-1β production in ciPTEC-OAT1. (**A**) Representative images (20×) and (**B**) quantification of p65 nuclear expression in ciPTEC-OAT1 after 1 h incubation with IS (200 μM, 1 mM, 2 mM), UT mix (1×, 2.5×), LPS (200 μg/mL) or TNF-α (100 ng/mL). (**C**) Relative mRNA expression of IL-1β upon 4 h exposure to either IS (200 μM, 1 mM, 2 mM) or UT mix (1×, 2.5×), in the absence or presence of specific IKK inhibitor BMS-345541 (4 μM) and compared to control (untreated ciPTEC-OAT1). (**D**) IL-1β secreted levels (pg/mL) by ciPEC-OAT1 following 24 h treatment with IS (200 μM, 1 mM, 2 mM) and UT mix (1×, 2.5×), in the absence or presence of BMS-345541 (4 μM). Data are derived from at least two independent experiments performed at least in duplicate and expressed as mean ± SEM. * *p* < 0.05, ** *p* < 0.01, *** *p* < 0.001, **** *p* < 0.0001 (One-way ANOVA followed by Dunnett’s multiple comparison test, using either untreated or PBUTs treated cells as a control).

NF-κB activation is usually required as a priming step in inflammasome-mediated IL-1β production. As an indication of NF-κB activation, nuclear translocation of p65 was assessed after exposing ciPTEC-OAT1 to PBUTs. Increased nuclear intensity of p65 was observed after all treatment conditions, including positive control treatments with either LPS or TNF-α (Figure 5A,B). As a further evidence of NF-κB activation, mRNA levels of some of the most relevant target genes were determined. Among those, IL-1β mRNA levels were significantly upregulated following 4 h exposure to IS and UT mix in the presence of the selective IKK inhibitor, BMS-345541 (Figure 5C). Comparable expression patterns were observed for other NF-κB target genes, *HMOX1*, *NFKBIA* and *TNF* (Figure S3A–C). Finally, IL-1β secretion was measured after co-treatment with PBUTs and BMS-345541. As shown in Figure 5D, all treatment conditions significantly increased IL-1β levels, while in the presence of the IKK inhibitor there was a significant reduction of IL-1β secretion.

### 3.6. Increased PBUTs Plasma Levels, Oxidative Stress and NLRP3 Inflammasome-Related Protein Expression in a CKD Rat Model

**Figure 6:**
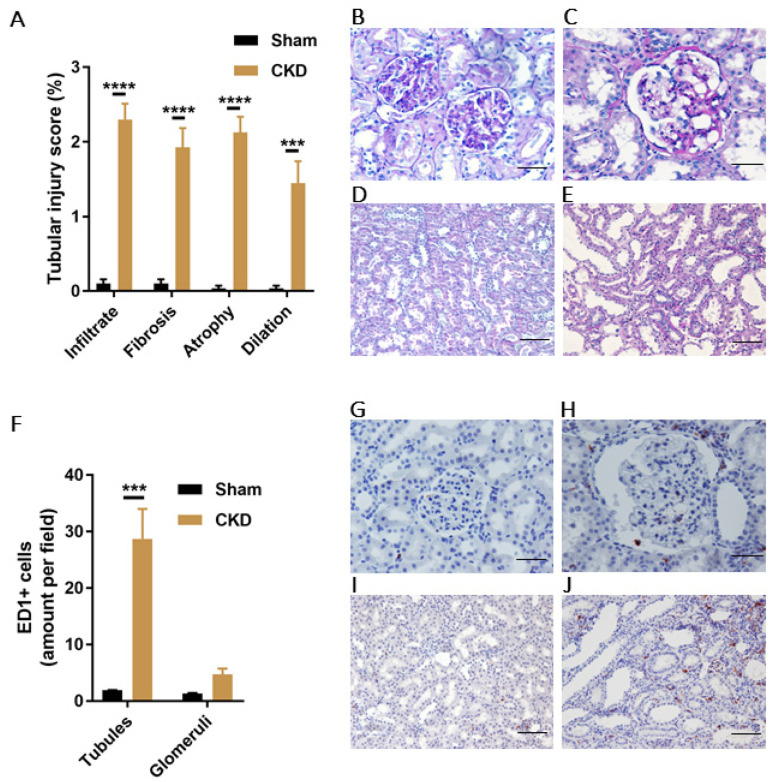
Characteristics of the CKD model in female Sprague Dawley rats created using single-procedure SNX. (**A**) Tubular injury score (%) in CKD and Sham rats, characterized by infiltrate, tubular fibrosis, atrophy and dilation. Representative PAS-staining images of glomeruli (20×) in (**B**) Sham and (**C**) CKD rats. Scale bars 50 μm. Representative PAS-staining images of tubules (10×) in (**D**) Sham and (**E**) CKD rats. Scale bars 100 μm. (**F**) Quantification of ED1 positive cells in tubules and glomeruli of Sham and CKD rats. Representative images of ED1 staining in glomeruli (20×) of (**G**) Sham and (**H**) CKD animals. Scale bars 50 μm. Representative images of ED1 staining in tubules (10×) of (**I**) Sham and (**J**) CKD animals. Scale bars 100 μm. Data are derived from *n* = 3 Sham and *n* = 4 CKD animals and presented as mean ± SEM. *** *p* < 0.001, **** *p* < 0.0001 (Two-way ANOVA followed by Sidaks’s multiple comparison test).

Our CKD rat model was created via single-procedure SNX in female Sprague Dawley rats. Prior to CKD induction, 9-weeks old rats in Sham and CKD groups showed similar baseline values for body weight and several CKD-related experimental parameters (Table 2). At approximately 5 months after SNX, the successful induction of CKD was confirmed by increased values of SBP, plasma creatinine and urea, and proteinuria in CKD compared with Sham. Progression to CKD was confirmed by histological analyses of the kidney remnants, both by high TI injury score (Figure 6A–E) and abundant presence of immune cells (macrophages, ED1^+^), especially in the TI area of CKD animals (Figure 6F–J).

**Figure 7:**
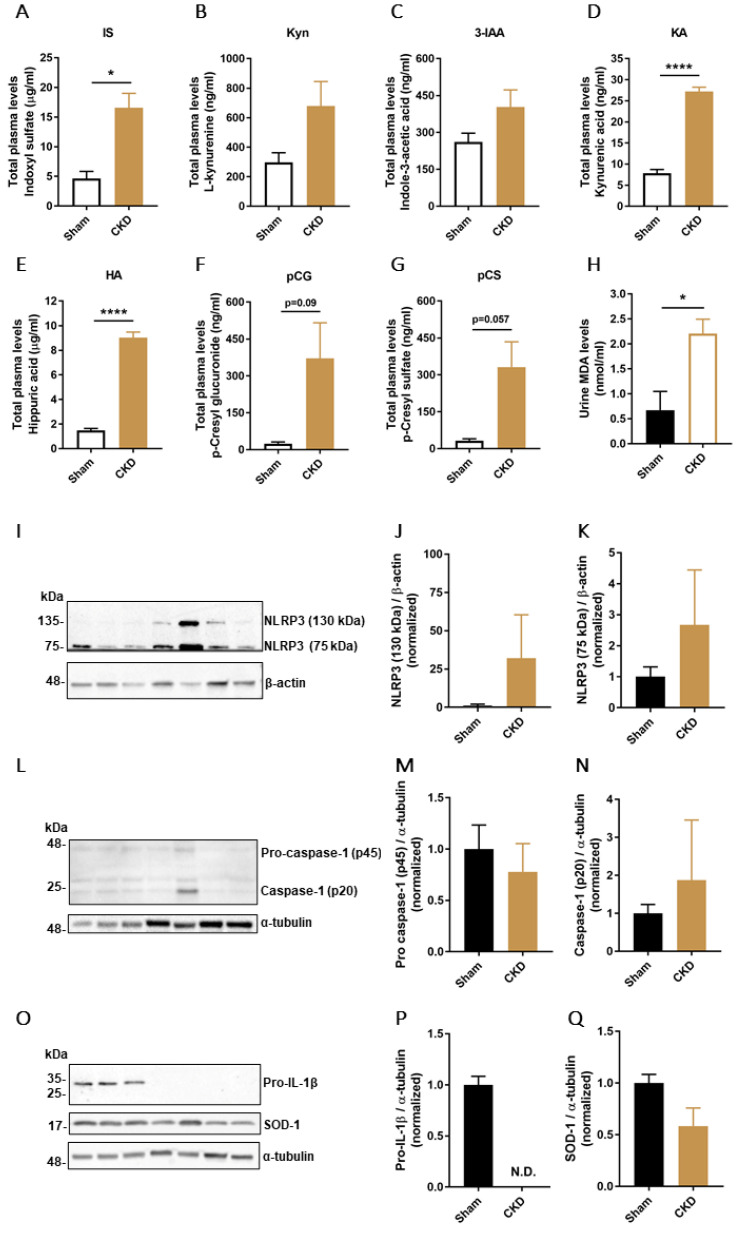
PBUTs plasma levels, oxidative damage and NLRP3 inflammasome components protein expression in CKD model. Quantification of total plasma levels (μg/mL or ng/mL) of (**A**) indoxyl sulfate (IS), (**B**) L-kynurenine (Kyn), (**C**) Indole-3-acetic acid (3-IAA), (**D**) kynurenic acid (KA), (**E**) hippuric acid (HA), (**F**) *p*-cresyl glucuronide (pCG) and **G**) *p*-cresyl sulfate (pCS) in Sham and CKD rats. (**H**) Urine MDA levels (nmol/mL) in Sham and CKD animals. (**I**–**K**) Representative Western blot and quantification of relative expression of NLRP3 in Sham and CKD rats; normalized to β-actin. (**L**–**N**) Representative Western blot and quantification of relative expression of pro-caspase-1 (p45) and caspase-1 (p20) in Sham and CKD rats; normalized to α-tubulin. (**O**) Representative Western blot of pro-IL-1β and SOD-1 in Sham and CKD rats. Quantification of relative expression of (**P**) pro-IL-1β and (**Q**) SOD-1; normalized to α-tubulin. Data are derived from *n* = 3 Sham and *n* = 4 CKD animals and presented as mean ± SEM. * *p* < 0.05, **** *p* < 0.0001 (Unpaired *t*-test).

Next, plasma levels of seven PBUTs were measured in both Sham and CKD animals (Figure 7A–G) and the results obtained showed a significant increase of IS, kynurenic acid, hippuric acid, and an increasing trend of L-kynurenine, indole-3-acetic acid, *p*-cresyl glucuronide and *p*-cresyl sulfate in the CKD group. Moreover, urine MDA levels, used as an indicator of CKD-associated oxidative damage, showed a 3.3-fold increase in the CKD group compared with Sham animals (Figure 7H).

To determine the effects of CKD on the inflammasome regulation in kidney tissue, protein levels of NLRP3, and inflammasome-related proteins pro-caspase-1 (p45), caspase-1 (p20), and pro-IL-1β were measured together with superoxide dismutase-1 (SOD-1) as an additional marker of oxidative stress. The results obtained show that there was an increasing trend of expression in kidney tissue of two different isoforms of NLRP3 in CKD animals (Figure 7I–K). An increasing trend for caspase-1 (p20) in the CKD animals and no difference in expression levels of pro-caspase-1 (p45) were found in the two groups (Figure 7L–N), while pro-IL-1β was detectable only in the Sham group (Figure 7O–P). Finally, the kidney levels of SOD-1 showed a strong decreasing trend in the CKD group compared to Sham (Figure 7O,Q).

## 4. Discussion

PBUTs are known pro-inflammatory and pro-oxidative metabolites whose negative consequences in the context of CKD are well-described [18,40]. IL-1β, a pro-inflammatory mediator known to have systemic consequences, is a marker of NLRP3 inflammasome activation and can promote kidney inflammation [41]. The NLRP3 inflammasome has also been shown to be involved in various CKD-related conditions, as demonstrated in different experimental models of kidney disease. For instance, reduced expression and maturation of IL-1β, IL-18 and caspase-1 activation and reduced tubular damage and fibrosis in an *NLRP3* knockout unilateral ureteral obstruction (UUO) model was observed [42]. The NLRP3 inflammasome inhibition has been also shown to ameliorate TI damage in a remnant kidney model [43]. Furthermore, various factors related to CKD such as proteinuria, aldosterone, calcinosis and mitochondrial dysfunction have been shown to directly interfere with inflammasome and subsequent kidney injury [44,45,46]. In addition, the NLRP3 inflammasome involvement has been described in other forms of kidney disease, including crystalline nephropathies, diabetic nephropathy, IgA nephropathy as well as lupus nephritis [26,47]. Finally, it has been demonstrated that NLRP3 inflammasome is activated in CKD patients undergoing dialysis treatment [48,49]. On the other hand, IS has been shown to influence IL-1β secretion during kidney injury in various cell types, including endothelial cells, macrophages and monocytes, via ROS, NF-κB and/or mitogen-activated protein kinase pathways [50,51,52,53]. However, the effects of PBUTs on IL-1β production by kidney epithelial cells, in particular proximal tubule cells, and the molecular mechanisms involved, are still unclear.

**Figure 8:**
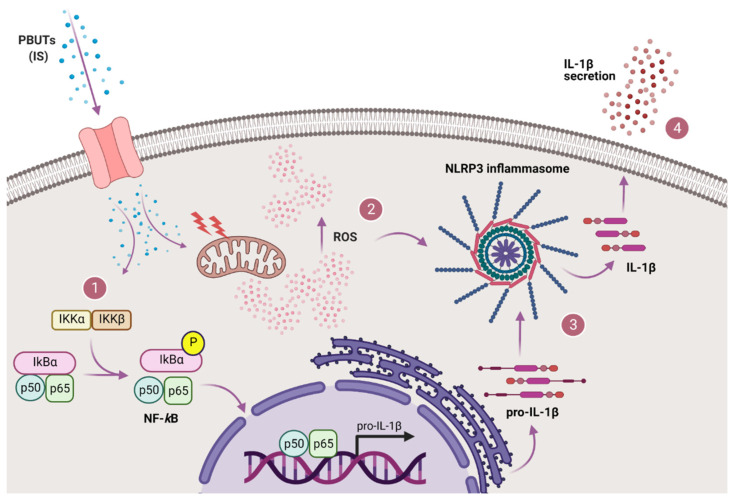
Proposed mechanism of PBUTs-ROS-driven NLRP3 inflammasome-mediated IL-1β production in proximal tubule epithelial cells. After reaching the cells, IS and other PBUTs lead to both (1) NF-κB signaling activation (p50/p65 translocation to the nucleus and transcription initiation) and (2) ROS production and oxidative stress. Following enhanced transcription of IL-1β and production of pro-IL-1β, ROS-mediated NLRP3 inflammasome is assembled thus leading to caspase-1 activation, and subsequent (3) proteolytic cleavage of pro-IL-1β into IL-1β that is (4) secreted by the cells. Created with BioRender.com.

In the present study, we demonstrated that PBUTs are capable of inducing inflammasome-mediated IL-1β production and secretion in human proximal tubule epithelial cells (ciPTEC-OAT1). Our results point towards a PBUT-mediated inflammasome activation, which is dependent on oxidative stress and activation of the NF-κB signaling pathway (summarized in Figure 8). Upon entering the cells via organic anion transporter 1 (OAT-1), or interacting with surface receptors, such as epidermal growth factor receptor (EGFR) [54,55], IS, and presumably other anionic PBUTs, stimulate NF-κB with subsequent p50/p65 translocation to the nucleus and transcription initiation, as well as ROS generation and oxidative stress. Following enhanced transcription of IL-1β, ROS-mediated NLRP3 inflammasome assembly leads to caspase-1 activation and subsequent proteolytic cleavage of pro-IL-1β into IL-1β that is secreted by the cells.

To our knowledge, we showed for the first time that IS (and the PBUTs mixture) induced NLRP3 expression both at mRNA and protein levels in proximal tubule cells, accompanied by increased caspase-1 activity and IL-1β expression and secretion. These findings are in accordance with a previously published study focusing on IS effects on rat myoblast cells in the context of cardiac contractile dysfunction associated with CKD, in which an upregulation of NLRP3, IL-1β and activation of NF-κB were observed [56]. However, conflicting effects of IS on NLRP3 inflammasome regulation have been reported as well. Wakamatsu et al. observed that NLRP3 expression was reduced in macrophages exposed to IS, whereas caspase-1 and IL-1β expression were increased [51]. Similarly, another study with macrophages showed that IS decreased NLRP3 protein expression, while increasing IL-1β secretion, in addition to showing that IS exposure prior to inflammasome induction suppressed caspase-1 maturation and NLRP3 expression [57]. Both these studies focused on THP-1-derived macrophages, in contrast to myoblasts and to our study which addressed tubular epithelial cells. Immune cells, including macrophages, different from other cell types, are specialized in orchestrating efficient activation of the inflammasome in response to danger signals [58]. This suggests that different cell types could present distinct responses to certain stimuli. Proximal tubular cells are specialized in the uptake and excretion of IS thanks to the abundant expression of transporters of the organic anion pathway, and therefore can be more susceptible to their biological effects compared to other cell types [30,59,60]. In fact, IS is directly cytotoxic to renal tubules and is considered as one of the most important toxins contributing to CKD progression [61]. The oxidative stress induced by IS, associated with a production of a plethora of pro-inflammatory and pro-fibrotic factors, is one of the key players of IS-mediated cytotoxicity of tubular cells [31,61]. Therefore, the contrasting observations on how IS influences NLRP3 inflammasome activation and the lack of relevant studies suggest that further research implementing different cell types and animal models is required to better understand the role of PBUTs in inflammasome regulation in specific tissues and organs. 

As mentioned earlier, inflammasome activation and subsequent IL-1β production requires two signals: the first enhances pro-inflammatory cytokines (IL-1β, IL-18) synthesis and the second triggers inflammasome formation [21,62]. We have shown that PBUTs are able to provide both signals. In line with literature describing the effects of IS on NF-κB pathway activation, we observed that IS induced p65 nuclear translocation and subsequent increase in target genes expression levels (*IL1B*, *HMOX1*, *NFKBIA* and *TNF*), confirming the ability of IS to provide the first signal in inflammasome activation [51,63]. Moreover, we showed that IS-mediated increase of intracellular ROS levels is also important in the regulation of IL-1β production, since the use of ROS inhibitor NAC abolished both caspase-1 activity and IL-1β secretion, suggesting its crucial role in inflammasome activation. This finding is in accordance with previous studies focusing on IS effects on redox imbalance and ROS production in various cell types [64,65,66].

Treatment with LPS and ATP augmented caspase-1 activity and IL-1β secretion in ciPTEC-OAT1, which proves that the cell model used in this study responds effectively to NLRP3 inflammasome-stimulating agents. Previous studies have shown that this particular PAMPs/DAMPs treatment combination is a very potent stimulus, as the pro-inflammatory LPS provides a priming step causing IL-1β upregulation via TLR4/NF-κB signaling and extracellular ATP provides a necessary triggering signal for NLRP3 inflammasome assembly and activation by inducing K^+^ efflux through interaction with purinergic P2X7 receptor [67,68]. In line with literature, LPS/ATP-treated ciPTEC-OAT1 responded to both the specific caspase-1 inhibitor ac-YVAD-cmk and the indirect inflammasome inhibitor glyburide with a decreased IL-1β production [69,70,71]. Consistently, ASC expression did not change on mRNA nor protein levels after 24 h exposure, while IL-1β and caspase-1 were both upregulated [62]. NLRP3, however, did show a slight increasing trend in protein levels, and downregulation on mRNA levels, potentially indicating the presence of a tissue (kidney) specific negative regulatory mechanism [72]. 

Finally, our SNX model presented typical characteristics of progressing CKD, including hypertension, proteinuria, uremia, as well as histological features reflecting tubular atrophy, fibrosis and abundant TI inflammation [37]. We showed a significant difference of several PBUTs plasma levels in CKD compared to Sham rats, as well as urine levels of MDA, clearly suggesting ongoing pro-inflammatory and oxidative status in these diseased animals. Moreover, the increased PBUTs plasma levels, urine MDA and low kidney SOD-1 correlate with the expression of some of the NLRP3 inflammasome-related markers as well. We observed strong tendencies for increased NLRP3 expression in CKD kidney tissue for two different molecular bands of NLRP3 with respect to healthy tissue, similar to what was observed for other CKD models [42]. While the expected size of NLRP3 is 118 kDa, most likely the high molecular band of approximately 130 kDa that we detected in kidney tissue is the result of a co-migration of the 118 kDa isoform and an isoform lacking two leucine-rich repeats (LRRs), whereas the low molecular band (75 kDa) is a short isoform lacking all the LRRs, and is prevalently derived from immune cells [73]. Moreover, it has been shown that the LRR domain of the NLRP3 is subject to alternative splicing that can be regulated on single cell level, and that can lead to NLRP3 increased functional diversity especially in the innate immune cells [74]. Comparable but less pronounced expression pattern was observed for activated caspase-1 (p20), while no expression of IL-1β could be detected. This is most likely due to the rapid secretion and depletion of the produced IL-1β given that the kidney is the main site of IL-1β metabolic degradation, in association also with a low half-life of the cytokine and possibly late time point used here for detection [75,76]. On the other hand, pro-IL-1β was only detected in healthy kidneys, suggesting that less IL-1β was produced in healthy rats compared to CKD rats in which a more enhanced IL-1β processing could have taken place. The expression levels of SOD-1 detected were lower in CKD compared to Sham rats which is in accordance with previous findings showing markedly reduced SOD-1 expression and superoxide scavenging activity (SOD activity equivalent) in CKD rats, further aggravated by IS [77]. For more conclusive findings, a larger number of animals and multiple time points for markers detection should be taken along in future studies. Studies involving knock out models for NLRP3 inflammasome and its components and/or PBUTs-relevant enzymes and transporters could greatly help understand better how PBUTs regulate NLRP3 inflammasome in proximal tubule cells and improve the comprehension of incongruous observations.

In conclusion, in the present study we showed for the first time that IS and PBUTs are involved in inflammasome-mediated IL-1β secretion by proximal tubule cells and that the most likely mechanism of NLRP3 inflammasome activation is the ROS model [29]. This suggests that the observed PBUTs-mediated interplay between pro-inflammatory and oxidative signaling pathways in the proximal tubule leads to IL-1β release, which could potentially be exploited for specific treatments aimed at contrasting CKD-associated inflammation. 

## Figures and Tables

**Table 1:** Concentrations of selected protein-bound uremic toxins (PBUTs) in healthy individuals, kidney disease patients and as applied within UT mixture in the present study ^1^.

Protein-Bound Uremic Toxin (PBUT)	Normal Concentrations (μM) (Mean ± SD)	Uremic Concentrations (μM) (Mean ± SD)	1× UT Mix (μM)	2.5× UT Mix (μM)
Indoxyl sulfate (IS)	2.3 ± 18.8	174 ± 122	100	250
L-kynurenine (Kyn)	1.9	3.3 ± 0.9	5	12.5
Indole-3-acetic acid (3-IAA)	2.9 ± 1.7	11.4 ± 2.3	3	7.5
Kynurenic acid (KA)	0.03 ± 0.01	0.8 ± 0.4	3	7.5
Hippuric acid (HA)	16.7 ± 11.2	608 ± 363	300	750
Indoxyl-β-glucuronide (IG)	3.1 ± 1.3	9.4 ± 9.4	10	25
*p*-cresyl glucuronide (pCG)	0.3 ± 0.2	30.1 ± 6.7	40	100
*p*-cresyl sulfate (pCS)	10.1 ± 12.2	122 ± 90	125	312.5

^1^ Concentrations reported and used are adapted from EUTox Uremic Solutes Database (https://www.uremic-toxins.org accessed on 19 August 2021) and Mihajlovic et al. [32].

**Table 2:** Main parameters (baseline and endpoint) evaluated in healthy (Sham; *n* = 3) and CKD (SNX; *n* = 4) animals ^1^.

Parameter	Baseline		Endpoint	
	Healthy (Sham)	CKD (SNX)	Healthy (Sham)	CKD (SNX)
Age (weeks)	9	9	29	31
Body weight (g)	174.3 ± 10.7	182.5 ± 7.0	276.0 ± 16.0 **	293.0 ± 18.6 ***
Systolic blood pressure (SBP) (mmHg)	120.7 ± 5.8	142.7 ± 16.0	128.0 ± 5.4	202.9 ± 18.8 *#
Hematocrit (%)	41.3 ± 0.9	40.5 ± 0.7	42.7 ± 0.9	41.3 ± 1.0
Plasma creatinine (µmol/L)	33.9 ± 8.6	29.1 ± 3.8	34.3 ± 5.6	77.1 ± 9.9 **#
Urine creatinine (µmol/24 h)	42.4 ± 1.2	39.2 ± 2.4	78.2 ± 10.7	89.1 ± 14.8 *
Plasma urea (mmol/L)	5.1 ± 0.3	4.9 ± 0.4	6.7 ± 0.6	16.3 ± 2.8 **#
Urine Na^+^ (µmol/24 h)	451.1 ± 88.2	386.4 ± 45.7	509.1 ± 48.3	540.9 ± 110.4
Urine K^+^ (µmol/24 h)	914.3 ± 81.6	773.9 ± 59.3	975.6 ± 133.0	1840.1 ± 353.7 *
Proteinuria (mg/24 h)	2.3 ± 0.5	2.3 ± 0.3	2.1 ± 0.1	329.4 ± 55.9 ***###

^1^ Age is presented as median, all the other parameters are presented as mean ± SEM. * *p* < 0.05, ** *p* < 0.01, *** *p* < 0.001 (Two-way ANOVA followed by Sidak’s multiple comparison test, using corresponding baseline values as controls); # *p* < 0.05, ### *p* < 0.001 (Two-way ANOVA followed by Sidak’s multiple comparison test, using corresponding Sham values as controls).

## Data Availability

All data generated or analyzed during this study are included in the published article.

## Supplementary Materials

The following are available online at https://www.mdpi.com/article/10.3390/biomedicines9101326/s1, Figure S1: Effect of LPS/ATP treatment on expression of NLRP3 inflammasome-related components in ciPTEC-OAT1, Figure S2: Effect of PBUTs-induced oxidative stress and antioxidant Trolox on IL-1β production in ciPTEC-OAT1, Figure S3: Effect of PBUTs on NF-κB target genes expression levels in ciPTEC-OAT1.
