# Supplementary material for: Protein-Bound Uremic Toxins Induce Reactive Oxygen Species-Dependent and Inflammasome-Mediated IL-1β Production in Kidney Proximal Tubule Cells
Source: Biomedicines. 2021 Sep 26;9(10):1326. doi: 10.3390/biomedicines9101326 (PMC8533138; doi:10.3390/biomedicines9101326)
Supplement: Supplementary file 1 [file biomedicines-09-01326-s001.zip › biomedicines-1370768-supplementary.pdf]

Supplementary Materials

Protein-bound uremic toxins induce reactive oxygen species-dependent and inflammasome-mediated IL-1β production in kidney proximal tubule cells

Milos Mihajlovic [1], Merle M. Krebber [2], Yi Yang [1], Sabbir Ahmed [1], Valeria Lozovanu [1], Daria Andreeva [1], Marianne C. Verhaar [2] and Rosalinde Masereeuw [1,*]

[1]Division of Pharmacology, Utrecht Institute for Pharmaceutical Sciences, Utrecht University, 3584 CG Utrecht, The Netherlands
[2]Department Nephrology and Hypertension, University Medical Center Utrecht, 3508 GA Utrecht, The Netherlands
*Correspondence: Prof. Dr. Rosalinde Masereeuw; e-mail: r.masereeuw@uu.nl

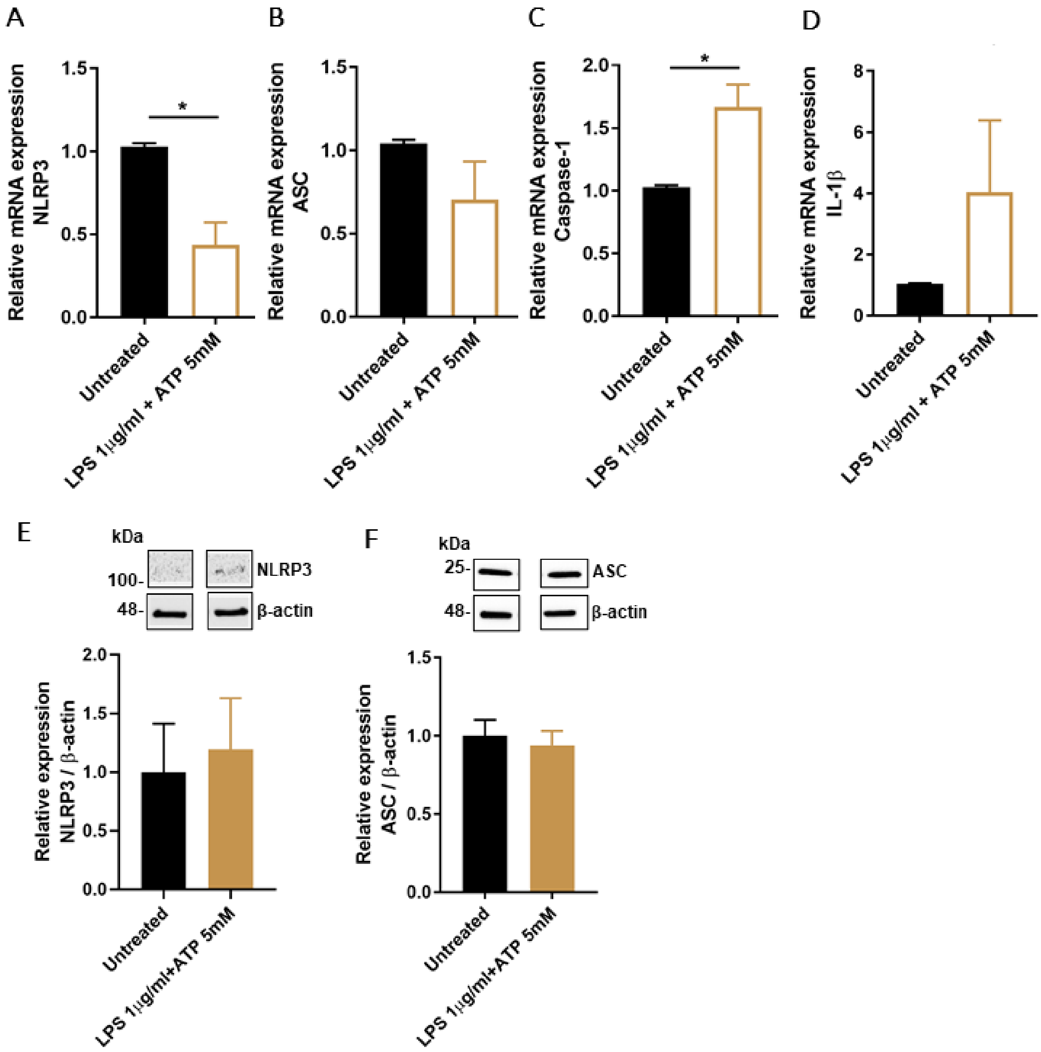

Figure S1. Effect of LPS/ATP treatment on expression of NLRP3 inflammasome-related components in ciPTEC-OAT1. Relative mRNA expression of **A**) NLRP3, **B**) ASC, **C**) caspase-1 and **D**) IL-1β upon exposure to LPS (1 μg/ml; 24 h) and ATP (5 mM; 30 min), compared to control (untreated ciPTEC-OAT1). Representative Western blot and quantification of relative expression of **E**) NLRP3 and **F**) ASC in ciPTEC-OAT1 following exposure to LPS (1 μg/ml; 24 h) and ATP (5 mM; 30 min); normalized to β-actin. Data are derived from three independent experiments performed in triplicate and expressed as mean ± SEM. * $p<0.05$ (Unpaired t test).

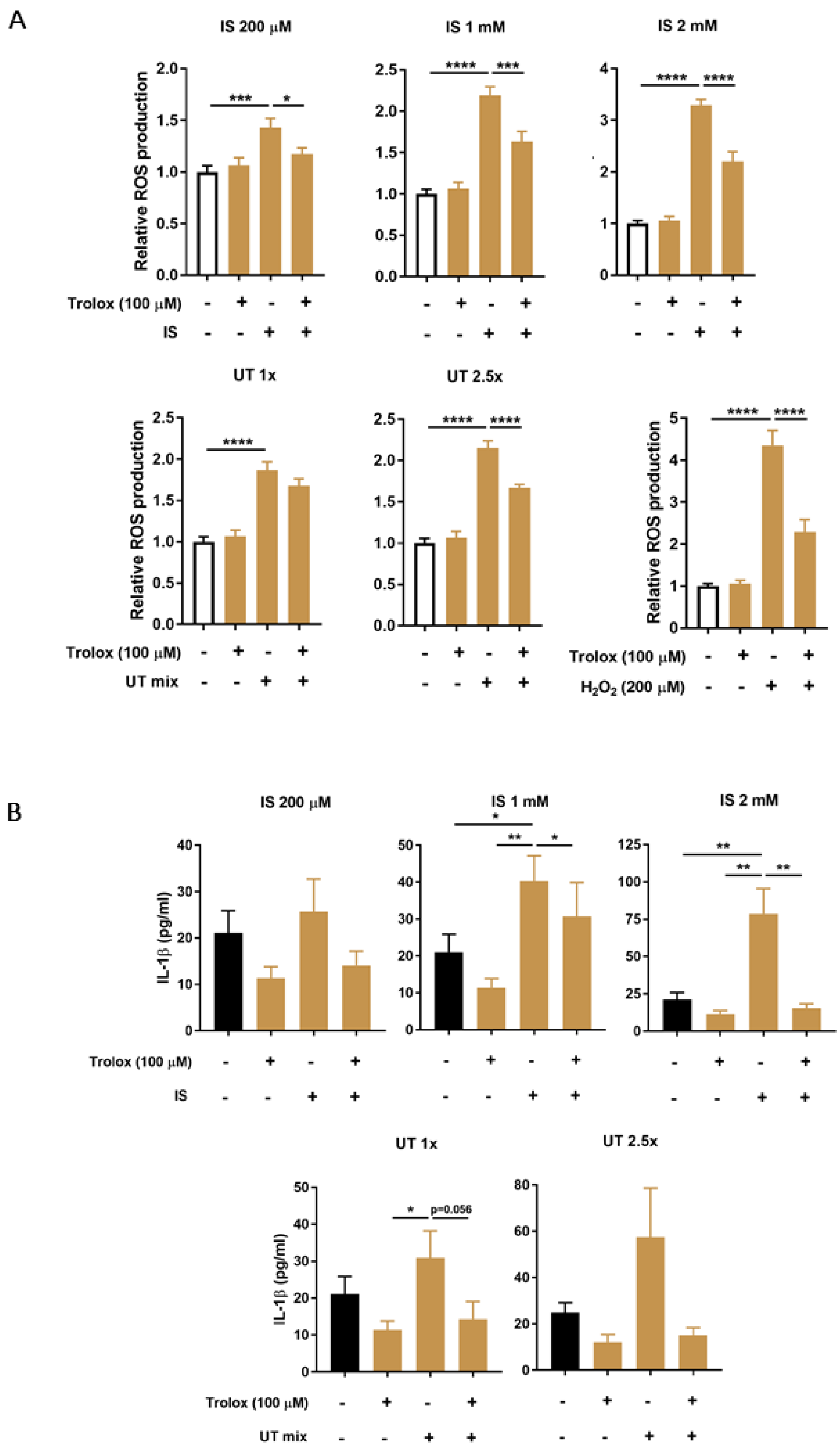

Figure S2. Effect of PBUTs-induced oxidative stress and antioxidant Trolox on IL-1β production in ciPTEC-OAT1. **A**) Relative intracellular ROS production in ciPTEC-OAT1 after 2 h exposure to IS (200 μM, 1 mM, 2 mM), UT mix (1x, 2.5x) and H_2O_2 (200 μM, positive control), in the absence or presence of ROS inhibitor Trolox (100 μM). **B**) IL-1β secreted levels (pg/ml) by ciPEC-OAT1 upon 24 h treatment with IS (200 μM, 1 mM, 2 mM) and UT mix (1x, 2.5x), in the absence or presence of Trolox (100 μM). Data are derived from three independent experiments performed in triplicate and expressed as mean ± SEM. * $p<0.05$, ** $p<0.01$, *** $p<0.001$, **** $p<0.0001$ (One-way ANOVA followed by Dunnett's multiple comparison test, using PBUTs treated cells as a control).

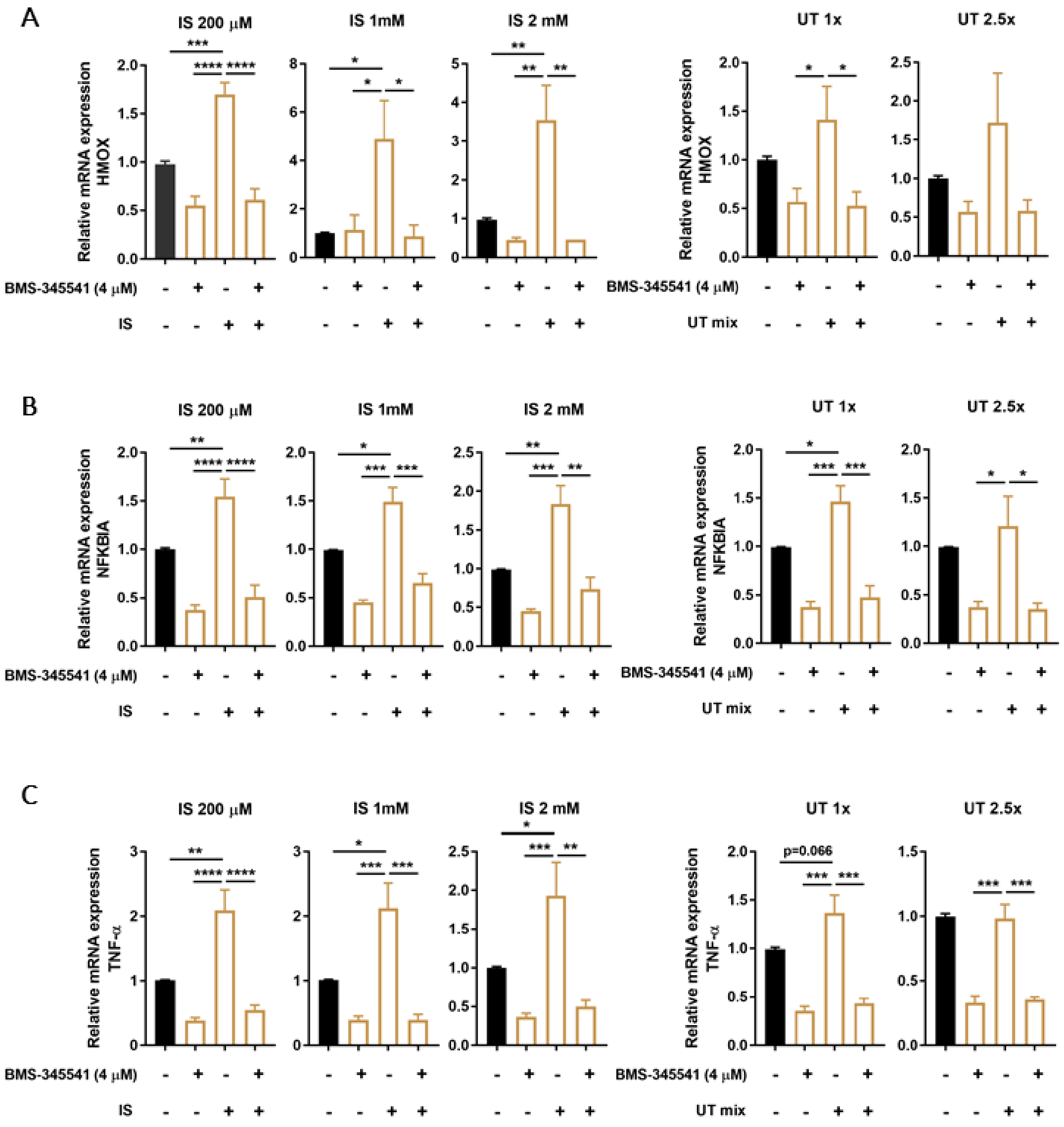

Figure S3. Effect of PBUTs on NF-κB target genes expression levels in ciPTEC-OAT1. Relative mRNA expression of **A**) heme oxygenase 1 (*HMOX1*), **B**) NFκB inhibitor alpha (*NFKBIA*) and **C**) TNF-*α* (*TNF*) upon 4 h exposure to either IS (200 μM, 1 mM, 2 mM) or UT mix (1x, 2.5x), in the absence or presence of specific IKK inhibitor BMS-345541 (4 μM) and compared to control (untreated ciPTEC-OAT1). Data are derived from three independent experiments performed in duplicate and expressed as mean ± SEM. * $p<0.05$, ** $p<0.01$, *** $p<0.001$, **** $p<0.0001$ (One-way ANOVA followed by Dunnett's multiple comparison test, using PBUTs treated cells as a control).
